# De-escalating breast and axillary surgery in breast cancer: evidence, controversies, and future directions

**DOI:** 10.3389/fonc.2026.1717340

**Published:** 2026-02-24

**Authors:** Baha Sharaf, Ala’ Seif, Fadi Alawneh, Hira Bani Hani, Marwa Sh Abrahim, Sharif Jehad, Mohamed Abdallah, Suleiman Mahafdah

**Affiliations:** 1 Department of Medicine, King Hussein Cancer Center, Amman, Jordan; 2 Department of Surgery, King Hussein Cancer Center, Amman, Jordan; 3 School of Medicine, Yarmouk University, Irbid, Jordan; 4 Department of Surgery, Jordanian Royal Medical Services, Amman, Jordan

**Keywords:** axillary management, breast cancer, deescalation, mastectomy, oncology surgery

## Abstract

The surgical management of breast cancer has achieved considerable improvement, shifting from radical resections toward increasingly conservative approaches driven by the advances in systemic therapy, precision imaging, and tumor biology. De-escalation strategies reducing the extent of surgery in the breast and axilla, aiming to minimize treatment-related morbidity (e.g., lymphedema, chronic pain, impaired mobility), while maintaining oncologic safety. This review synthesizes evidence from over 20 landmark trials, critically examines current clinical guidelines, and explores emerging technologies (e.g., artificial intelligence, liquid biopsy) that may further refine patient selection. We also highlight global disparities in adopting de-escalation strategies and discuss unanswered questions, such as applicability in high-risk subtypes and long-term recurrence risks.

## 1 Introduction

The historical approach to breast cancer surgery was rooted in William Halsted’s radical mastectomy (RM) (1), involving *en bloc* removal of the breast, pectoral muscles, and axillary nodes, became the gold standard after improving 5-year survival from 13% to 40% (2) and was performed on more than 90% of patients until 1970 (3), With local and regional recurrence rates of 6% and 22% (4). However, it carried severe morbidity, with a regional recurrence rate of 22% despite extensive resection. More than 50% of patients developed chronic lymphedema, and many experienced significant chest wall deformities and shoulder dysfunction.

Patey and Madden’s (5–7) modifications preserved the pectoralis major, reducing morbidity while maintaining oncologic outcomes.

However, the Fisher (8) game changer in the 1980s redefined breast cancer as a systemic disease, reducing the necessity for aggressive local therapy in all patients.

This conceptual shift was later reinforced by clinical evidence from NSABP B-18, which demonstrated that neoadjuvant chemotherapy (NAC) achieved tumor shrinkage in 80% of 1, 523 operable breast cancer patients, enabling 12% more lumpectomies compared to adjuvant therapy. With 36% achieving complete remission, this landmark study proved for the first time that NAC could safely facilitate breast-conserving surgery (BCS) without compromising survival outcomes (9). Later on, the NSABP B-27 trial (2006) revolutionized neoadjuvant therapy by demonstrating that adding docetaxel to AC chemotherapy doubled pCR rates from 13% to 26% in 2, 411 breast cancer patients. This study not only confirmed the survival benefit of taxanes (DFS HR 0.71) but also established that achieving pCR predicted better long-term outcomes, solidifying taxane-based regimens as the standard for optimizing both tumor response and breast-conserving surgery eligibility (10).

## 2 Long-term outcomes of breast-conserving surgery versus mastectomy

Decades of clinical trials have consistently demonstrated equivalent survival outcomes between breast-conserving surgery (BCS) with radiotherapy and mastectomy (11–14). The Gustave-Roussy trial (15) (15-year follow-up) showed comparable overall survival rates of 73% for BCS versus 65% for mastectomy. In comparison, the landmark NSABP B-06 and Milan trials (20-year follow-up) confirmed this equivalence with 46-59% survival for BCS compared to 47-58% for mastectomy (16, 17). While early studies showed slightly higher local recurrence rates with BCS (9-14%) versus mastectomy (2-10%), these differences fell within acceptable oncologic thresholds (<5% at 5 years). Importantly, modern advances, including precision radiotherapy techniques and the adoption of “no tumor on ink” margin standards, have dramatically improved outcomes, reducing BCS recurrence rates to less than 2% in contemporary practice. These long-term data underscore that when performed with current surgical and radiation techniques, BCS provides equivalent cancer control to mastectomy while preserving the breast (**Table 1**) (15–17).

**Table 1:** Long-term outcomes of breast-conserving surgery versus mastectomy.

Trial (year)	Follow-up	OS (BCS+RT)	OS (mastectomy)	Local recurrence (BCS)	Local recurrence (mastectomy)
Gustave-Roussy (1996) (15)	15 yrs	73%	65%	13%	18%
NSABP B-06 (2002) (16)	20 yrs	46%	47%	14%	10%
Milan (2002) (17)	20 yrs	59%	58%	9%	2%

## 3 BCS in multicentric disease

The ACOSOG Z11102 trial (18) challenged conventional wisdom by evaluating breast-conserving surgery in 198 patients with multiple ipsilateral tumors (96% with two foci, median size 1.5 cm). The results were compelling: surgeons achieved clear margins in a single operation for 67.5% of patients, while only 7.1% required conversion to mastectomy. Most importantly, the 5-year local recurrence rate of 3.3% matched outcomes seen in single-tumor cases, demonstrating comparable oncologic safety. A critical finding was the dramatic impact of preoperative MRI, which slashed recurrence rates from 22.6% to just 1.7%, a statistically significant reduction (HR 13.49, p=0.02), highlighting MRI’s essential role in proper patient selection and surgical planning for multifocal disease (19). The 2020 follow-up analysis of the ACOSOG Z11102 trial provided valuable insights into patient-reported outcomes following breast-conserving surgery (BCS) for multifocal disease. Using validated BREAST-Q measures, researchers found remarkable stability in patient satisfaction scores, which only slightly declined from 76.3 points postoperatively to 73 points at 3-year follow-up. More encouragingly, patients’ sense of well-being showed substantial improvement over time, rising from 58 points immediately after surgery to 70–80 points by 5 years, reflecting meaningful recovery from initial treatment burdens. On the standardized 4-point Winchester & Cox scale, an overwhelming 82% of patients rated their cosmetic results as “good” or “excellent” at the 3-year mark. These outcomes proved comparable to those achieved with unifocal BCS in historic trials like NSABP B-06 (78% satisfaction at 5 years), confirming that multifocal BCS can successfully balance oncologic rigor with preservation of quality-of-life and body image - a crucial dual priority in contemporary breast cancer management (20) (**Table 2**)/.

**Table 2:** Comparative outcomes of breast-conserving surgery and mastectomy across retrospective cohort studies.

Study	Breast conserving surgery	Mastectomy	Conclusion
Nos et al. (1999) (21)	56	132	No significant difference in the recurrence or survival rates
Kaplan et al. (2003) (22)	36	19	The type of surgery had no impact on 5-year overall or disease-free survival.
Oh et al. (2006) (23)	20	27	No significant difference in disease-free or overall survival
Gentilini et al. (2009) (24)	476	–	Local recurrence rate of 5.1% at 5 years
Lynch et al. (2013) (25)	1757 UFBC	1059 UFBF	Breast-conserving surgery is a safe option for MFBC
Wolters et al. (2013) (26)	256 MFBC	417 MFBC	All MCBC had a mastectomy in this cohort
	623 MFBC	319 MFBC	No significant difference in disease-free or overall survival
	60 MCBC	40 MCBC	
Winters et al. (2018) (27)	3537 MFMCBC	–	Breast-conserving surgery and mastectomy had similar loco-regional recurrence for MFMCBC. The conclusion was to support a future randomized trial.

Across retrospective and prospective cohorts, BCS demonstrated comparable survival and recurrence outcomes to mastectomy in selected patients with multifocal or multicentric disease. When negative margins can be achieved and cosmetic outcomes maintained, breast conservation is a safe option.

### 3.1 Technical innovations

Oncoplastic & Extreme Conservation. The “extreme oncoplasty” cohort (86 MF/MC patients; median tumor span ~65 mm): Reported a local recurrence rate of 3.4% after ~75 months and excellent cosmetic results in nearly half of patients. Trials: Toward Definitive Answers. The UK MIAMI trial (28) is currently randomizing MF/MC patients to therapeutic mammoplasty (a form of oncoplastic BCS) versus mastectomy. There is no evidence that the surgical approach affects prognosis, provided that tumors are completely excised. Current evidence points to breast conservation as being safe for multifocal multicentric breast cancer (when technically feasible and provided that acceptable cosmetic results can be achieved). While some patients may opt for mastectomy over breast conservation, their choice should not be limited by a lack of surgical skill and local expertise in oncoplastic breast surgery.

## 4 Pathologic complete response and surgical omission (29)

In their 2025 narrative review entitled *“Omission of breast surgery in exceptional responders after neoadjuvant chemotherapy — what are future possibilities?”*, Phang and Weiss examine whether surgery can safely be omitted in patients with early-stage Triple Negative Breast Cancer (TNBC) or HER2-positive breast cancer (HER2+) who achieve a pathological complete response (pCR) following neoadjuvant chemotherapy (NAC).

They reviewed 49 studies published between 1989 and 2024, including clinical trials, retrospective analyses and review articles. Their analysis confirms that while TNBC and HER2+ tumors often respond very well to systemic therapy — with reported breast pCR rates up to ~64% (TNBC) and ~66% (HER2+) — reliably predicting pCR remains challenging.

Conventional physical examination and imaging (mammography, ultrasound, MRI) prove insufficient: in many cases they fail to detect residual disease accurately. Minimally invasive sampling methods, such as vacuum-assisted biopsy (VAB), especially when optimized (using adequate needle gauge and sufficient number of cores), show more promise: in selected, well-sampled cases false-negative rates (FNR) as low as ~2.9% have been reported.

Despite these encouraging findings, the authors emphasize that current “surgery-omission” trials are small, and their patient populations are highly selected. They also note a reluctance among many patients to skip surgery, even when medically feasible. As such, completed evidence is insufficient to recommend surgery omission broadly — including for multifocal or multicentric (MF/MC) tumors, where data remain especially scarce.

The authors conclude that while surgical de-escalation (most realistically, avoidance of mastectomy rather than complete omission of surgery) may become a valid option in future, larger-scale studies with long-term follow-up are needed. They also urge that patient preferences and resource constraints be considered carefully.

## 5 MICRA and RESPONDER trials

The MICRA trial (30) it is a multicenter, prospective single-arm study in three Dutch hospitals. Patients with T1-4(N0 or N +) breast cancer with MRI rPR and enhancement ≤ 2.0 cm or MRI rCR after NST were enrolled. Eight ultrasound-guided 14-G core biopsies were obtained in the operating room before surgery close to the marker placed centrally in the tumor area at diagnosis, Between April 2016 and June 2019, 202 patients fulfilled eligibility criteria. Pre-surgical biopsies were obtained in 167 patients, of whom 136 had rCR and 31 had rPR on MRI. Forty-three (26%) tumors were hormone receptor (HR)-positive/HER2-negative, 64 (38%) were HER2-positive, and 60 (36%) were triple-negative. Eighty-nine patients had pCR (53%; 95% CI 45-61) and 78 had residual disease. Biopsies were false-negative in 29 (37%; 95% CI 27-49) of 78 patients. The multivariable associated with false-negative biopsies was rCR (FNR 47%; OR 9.81, 95% CI 1.72-55.89; p = 0.01); a trend was observed for HR-negative tumors (FNR 71% in HER2-positive and 55% in triple-negative tumors; OR 4.55, 95% CI 0.95-21.73; p = 0.058) and smaller pathological lesions (6 mm vs 15 mm; OR 0.93, 95% CI 0.87-1.00; p = 0.051). The MICRA trial showed that ultrasound-guided core biopsies are not accurate enough to identify breast pCR in patients with good response on MRI after NST. Therefore, breast surgery cannot safely be omitted relying on the results of core biopsies in these patients.

The RESPONDER trial (NCT02945579) is an ongoing, multicenter phase II investigation designed to determine whether breast surgery can be omitted in patients with triple-negative or HER2-positive breast cancer who demonstrate a pathologic complete response (pCR) after neoadjuvant systemic therapy, including immunotherapy. To verify pCR, patients undergo image-guided vacuum-assisted core sampling of the tumor bed, with a minimum of 12 cores required to rule out residual disease. Individuals with biopsy-confirmed pCR may proceed directly to whole-breast radiotherapy, whereas those with persistent invasive or *in situ* carcinoma continue with standard surgical management.

The study also incorporates liquid-biopsy–based surveillance, including circulating tumor DNA (ctDNA) and circulating tumor cell (CTC) analyses, alongside imaging follow-up and patient-reported outcomes. Early results from the first 50 participants indicate that 62% demonstrated no remaining invasive or *in situ* cancer on post-neoadjuvant biopsy, and none of these patients experienced ipsilateral breast recurrence at a median follow-up of 26 months. Biopsy procedures were well tolerated, with no significant complications reported. These preliminary observations suggest that, within a carefully selected cohort, omission of breast surgery may be feasible without compromising early oncologic safety, and that ctDNA monitoring may serve as an additional safeguard against early relapse. Longer-term follow-up will be essential to confirm sustained local control and validate this strategy as a potential practice-changing approach for highly responsive disease subtypes (31, 32).

Parallel efforts have highlighted the challenges of reliably confirming pCR using percutaneous biopsy alone. The NRG-BR005 study (33), for example, evaluated the diagnostic performance of stereotactic tumor-bed sampling in patients who achieved both clinical and radiologic complete or near-complete responses following neoadjuvant chemotherapy. Eligibility required completion of neoadjuvant therapy and demonstration of cCR accompanied by stringent radiologic criteria—residual tumor bed ≤1 cm without malignant calcifications on mammography, ≤2 cm on ultrasound, and no suspicious enhancement on MRI. Participants underwent clip-directed stereotactic biopsies prior to breast-conserving surgery.

Among 98 evaluable patients with clinical T1–3 disease across breast cancer subtypes, 36 harbored residual disease at surgery. Only 18 of these cases were detected by stereotactic biopsy, yielding a sensitivity of 50% and an NPV of 77.5%, falling short of the predefined 90% threshold required to support surgery omission. These findings underscore the limitations of current biopsy techniques for confidently excluding residual disease. The final peer-reviewed report of NRG-BR005 is pending. Meanwhile, the I-SPY2 platform continues to advance neoadjuvant drug development by integrating novel therapeutics earlier in treatment, thereby increasing pCR rates and creating a stronger foundation for future trials exploring surgical de-escalation in triple-negative and HER2-positive breast cancer.

## 6 Future directions

The future of patient selection for multifocal breast-conserving surgery (BCS) is evolving with advances in genomic profiling, breast imaging, and more use of oncoplastic surgical techniques. Trials like NRG-BR008 are investigating whether genomic risk scores—based on each tumor’s biological signature can guide decisions between BCS and more extensive surgery (34). This approach builds on tools like Oncotype DX, focusing specifically on multifocal disease. Researchers aim to predict:

- Margin clearance success.
- Presence of occult lesions.
- Tumor aggressiveness across foci.

Early findings suggest genomic profiling may resolve current gray areas, especially in intermediate-risk cases where MRI is inconclusive. If validated, this strategy could reduce unnecessary mastectomies and enable safer, personalized breast conservation.

## 7 De-escalation of axillary surgery

**Figure 1:**
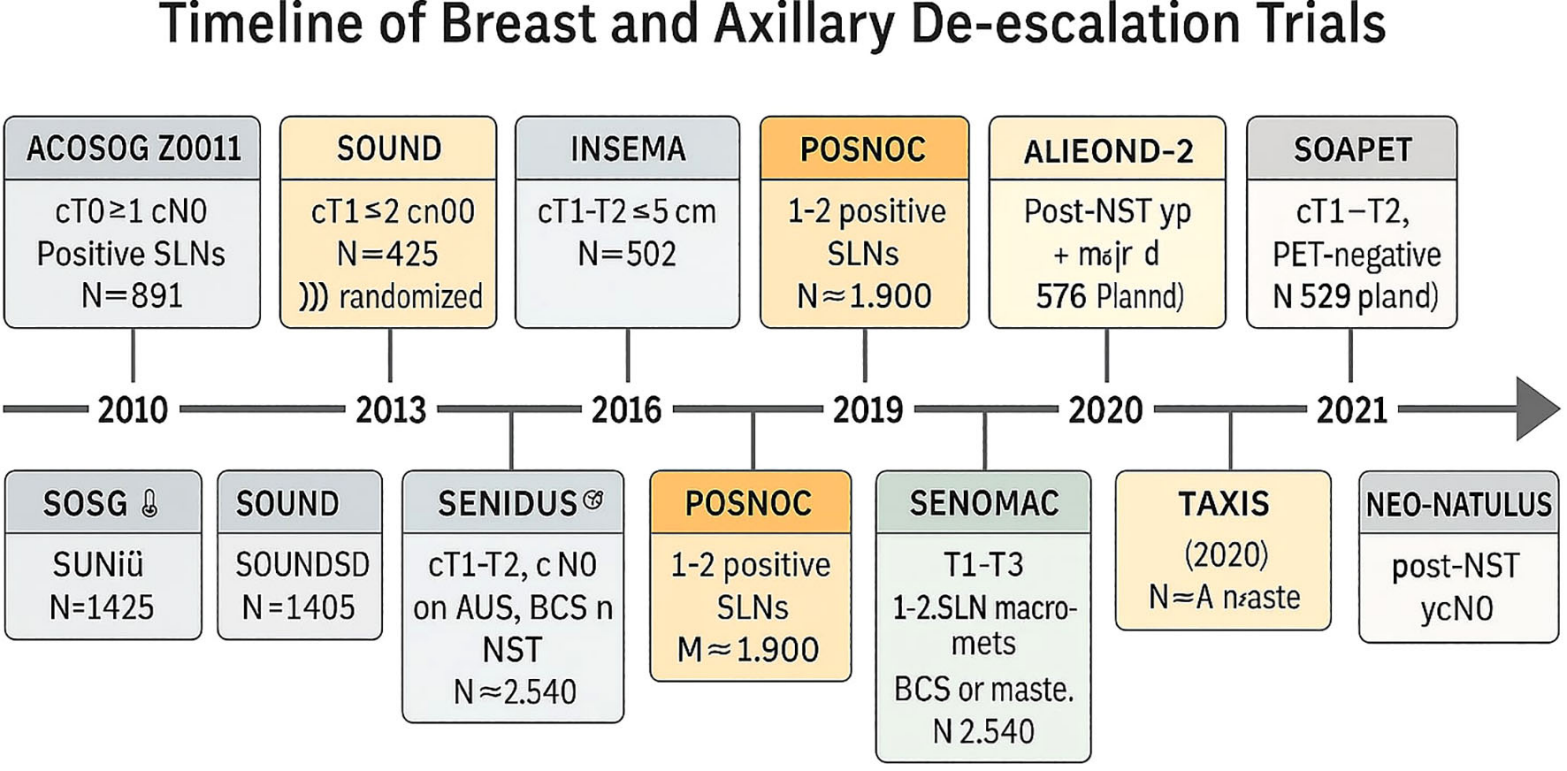
Timeline of breast and axillary de-esclation trials.

De-escalation of axillary surgery began with Giuliano’s introduction of sentinel lymph node biopsy (SLNB), which replaced axillary lymph node dissection (ALND) for clinically node-negative (cN0) patients, significantly reducing lymphedema rates from 25% to less than 5% (35). The NSABP B-32 trial confirmed the non-inferiority of SLNB, showing comparable 10-year overall survival to ALND (87% vs. 89%) (36). Modern strategies for de-escalation are driven by the effectiveness of systemic therapies in managing micro metastatic disease, advancements in preoperative imaging, and a focus on patient-reported outcomes such as lymphedema and quality of life. However, real-world adoption remains variable due to surgeon preferences, institutional practices, and access to genomic tools (**Figure 1**).

### 7.1 Omitting sentinel lymph node biopsy in clinically node-negative patients

#### 7.1.1 SOUND trial: ultrasound-based selection

The SOUND trial (Gentilini et al., 2021) randomized 1, 560 patients with T1 tumors (≤2 cm) and ultrasound-confirmed node-negative disease to SLNB or no SLNB. At 5 years, there was no difference in disease-free survival (98.7% vs 98.0%) or axillary recurrence (0.5% vs 0.4%) with Lymphedema reduction: 22% lower in the omission arm (37, 38).

A key limitation is the reliance on axillary ultrasound, which has a false-negative rate of ~25% in unselected patients. Additionally, the trial excluded lobular carcinoma and multifocal disease, limiting generalizability.

Clinical Impact:

- Validated SLNB omission in ultra-low-risk T1N0 patients with rigorous imaging.

#### 7.1.2 INSEMA trial

As the largest randomized study of its kind (N = 5, 940), the INSEMA trial employed an innovative two-phase design to evaluate axillary de-escalation in patients with tumors up to 5 cm. The study first randomized women to either sentinel lymph node biopsy (SLNB) or observation, then further randomized SLNB-positive patients to either axillary lymph node dissection (ALND) or no further surgery. The trial yielded practice-changing results (39, 40).

Equivalent oncologic outcomes: 5-year disease-free survival showed no significant difference between arms (HR 0.94, p=0.62), challenging the necessity of routine SLNB

Superior patient experience: Omitting SLNB resulted in a remarkable 30% reduction in arm morbidity, including lymphedema and mobility restrictions.

Broad clinical applicability: By including both T1 and T2 tumors (≤5 cm), the findings extend to a wider patient population than previous studies

This landmark trial provides the strongest evidence to date that many patients with early-stage breast cancer can safely avoid axillary surgery when systemic therapy is planned, significantly reducing treatment-related complications without compromising cancer outcomes. The results particularly support omitting SLNB in hormone receptor-positive, HER2-negative cases where adjuvant therapy decisions are primarily driven by tumor biology rather than nodal status.

#### 7.1.3 Practice change

Practice Change: Supports considering SLNB omission in well-imaged, low-risk (often HR+/HER2–) T1–T2 (≤5 cm) tumors when adjuvant systemic therapy is already planned; however, decisions should account for operator variability in axillary US, limited long-term follow-up, potential impact on systemic therapy selection, and the nodal-status-based eligibility for adjuvant CDK4/6 inhibitors (e.g., monarchE).

#### 7.1.4 NAUTILUS trial (Korea, 2020–2022)

This groundbreaking prospective study specifically evaluated axillary management strategies in 1, 734 Asian breast cancer patients with tumors up to 5 cm, all receiving standardized adjuvant systemic therapy and whole-breast radiotherapy. The trial’s design addressed an important gap in existing evidence. Asian populations typically demonstrate different tumor biology (including higher prevalence of HER2-positive subtypes) compared to Western cohorts studied in previous trials.

The results (41) provided compelling evidence for practice change with Excellent disease control: 3-year invasive disease-free survival was nearly identical between arms (97.1% without SLNB vs. 96.8% with SLNB). Minimal axillary recurrence: Both groups maintained exceptionally low 0.3% axillary recurrence rates.

Biological consistency: Outcomes remained stable across different molecular subtypes

As the first major trial to confirm the safety of SLNB omission in Asian patients, NAUTILUS carries particular significance for global practice guidelines. The findings demonstrate that genomic risk (rather than ethnicity) should drive axillary management decisions, supporting the feasibility of omitting SLNB in carefully selected patients regardless of racial background when modern systemic therapies are employed. This trial importantly expands the applicability of axillary de-escalation strategies to more diverse patient populations worldwide.

Caveats and generalizability. Although contemporary randomized data suggest that omission of axillary staging can be safe in carefully selected early-stage patients, the evidence base predominantly reflects HR+/HER2–, postmenopausal, early-stage populations. In most studies, axillary assessment depended on clinical examination and axillary ultrasound, both of which are operator- and institution-dependent, introducing variability in staging accuracy. Follow-up remains relatively short in several trials, and how omission of axillary staging influenced adjuvant systemic therapy selection is insufficiently detailed. These issues are increasingly relevant given the emerging adjuvant use of CDK4/6 inhibitors, where eligibility, as in monarchE (42), depends on nodal status (≥4 positive nodes or high-risk 1–3 nodes). Routine omission of SLNB may therefore risk under-staging patients who could qualify for escalated adjuvant therapy.

Recent systemic therapy trials such as RxPONDER (43) [Kalinsky et al., NEJM 2021] and TAILORx (44) demonstrated that adjuvant chemotherapy decisions in HR+/HER2– disease are increasingly guided by genomic assays rather than nodal status. This reinforces the argument that axillary staging may become redundant in many cases, as systemic treatment allocation is uncoupled from axillary findings. However, this also underscores the danger of under-staging patients who might qualify for escalated regimens such as adjuvant CDK4/6 inhibitors (monarchE) or novel immunotherapy combinations.

#### 7.1.5 The BOOG 13–08 trial

The BOOG 13–08 trial (45) was a prospective, randomized, phase III study that investigated the safety of omitting axillary lymph node dissection (ALND) in clinically node-negative (cN0) breast cancer patients who were found to have 1–3 positive sentinel lymph nodes (SLNs) after neoadjuvant systemic therapy (NAST).

The Dutch BOOG 13–08 study with the longest planned follow-up (10 years) specifically examines regional recurrence patterns in patients with T1-2N0 disease undergoing breast-conserving surgery with whole-breast radiotherapy. This rigorous design addresses a crucial evidence gap regarding the durability of omitting sentinel lymph node biopsy (SLNB) in early-stage patients.

#### 7.1.6 Early results at 5 years demonstrate

- Nearly identical regional control: 0.8% recurrence without SLNB versus 0.6% with SLNB
- Consistency with international data: These findings align with SOUND and INSEMA trial outcomes

BOOG 13-08’s comprehensive design provides critical reassurance about the long-term safety of axillary de-escalation strategies, particularly for luminal A tumors. These typically indolent but potentially late-recurring subtypes represent the majority of early breast cancer cases. The mature data will help establish whether the excellent early control rates persist through the peak recurrence risk period for luminal subtypes.

#### 7.1.7 SOAPET trial (China, 2019–ongoing)

The SOAPET trial (46) represents a significant advancement in the field of axillary staging, offering a potential paradigm shift from surgical to imaging-based assessment. This Chinese study has adopted a meticulous two-phase approach, first rigorously validating PET-CT’s impressive 98% negative predictive value for nodal staging, then prospectively evaluating whether SLNB can be safely omitted in PET-CT-negative patients. The implications of this research are particularly profound for resource-limited settings where PET-CT availability exceeds nuclear medicine capabilities required for traditional SLNB.

This innovative approach could fundamentally transform clinical practice by reducing surgical morbidity while maintaining staging accuracy. The trial’s potential impact is especially relevant for triple-negative and HER2-positive tumors, which typically demonstrate higher PET-CT avidity. However, several practical considerations temper immediate enthusiasm - current PET-CT accessibility constraints, cost-effectiveness compared to ultrasound-based methods, and varying performance across tumor subtypes all present challenges to widespread implementation (**Table 3**).

**Table 3:** Key landmark trials on axillary surgery de-escalation.

Study-	Patients (n)	Key inclusion criteria	Treatment arms	Median follow-up	Primary endpoint	Key clinicopathologic findings	Additional non-SLN positive nodes (ALND arm)	Prognostic results/clinical caveats
-ACOSOG Z0011	891	cT1–2, cN0; breast-conserving surgery + whole-breast RT; 1–2 positive SLNs	SLNB alone vs completion ALND	9.3 years	Overall survival (non-inferiority)	Low axillary recurrence with SLNB alone; long-term OS excellent	~27% had additional positive non-SLNs detected by ALND	10-yr OS: 86.3% (SLNB) vs 83.6% (ALND); Supports omission of routine ALND in selected patients (BCS + WBI). Limited direct data for aggressive subtypes or mastectomy.
EORTC AMAROS (10981-22023)	1, 425	cT1–2, cN0 with positive SLN	Axillary radiotherapy (ART) vs ALND	10 years	Axillary recurrence/locoregional control	Both arms achieved very low axillary recurrence; lymphedema significantly lower with ART	~33% additional positive nodes in ALND arm (trial reports variable rates)	10-yr axillary recurrence: 0.93% (ALND) vs 1.82% (ART); No OS or DFS difference. Lymphedema ~11.9% (ART) vs ~24–25% (ALND). ART preferred when axillary treatment is indicated.
SINODAR-ONE	891	cT1–2, cN0, 1–2 positive SLNs (included selected mastectomy patients)	SLNB alone vs ALND	Short (~34–40 months median; early reports ~3 yrs)	OS/recurrence	Very low event rates: 1 axillary recurrence per arm in early analyses; 5-yr OS ~98.8–98.9%	Additional positive non-SLNs present in ALND arm (proportion varies)	Promising short-term outcomes, but follow-up substantially shorter than Z0011/AMAROS; interpret cautiously, especially for mastectomy patients and high-risk biology.
OTOASOR (Optimal Treatment of the Axilla)	474	pT <3 cm, cN0, pN1(sn)	Regional nodal irradiation (RNI) vs ALND	8 years	8-yr axillary recurrence/OS	Comparable axillary control with low recurrence in both arms	Additional nodes reported in ALND arm	8-yr axillary recurrence: ALND 2.0% vs RNI 1.7%; no significant OS/DFS difference. Supports RNI as an alternative axillary treatment in selected patients.
SENOMAC (interim/*post-hoc* analyses)	2, 766 (randomized)	T1–T3, 1–2 positive SLNs; broader inclusion than Z0011 (including mastectomy)	SLNB alone vs completion ALND	Interim analyses; follow-up variable	Non-inferiority for recurrence/safety	Early/*post-hoc* analyses show no clear increase in recurrence with SLNB alone; lower lymphedema	Additional non-SLN disease present in ALND group in many patients	Large modern randomized study with broad criteria; early results support ALND omission in many patients, but final long-term oncologic outcomes are still maturing—important for generalizability beyond classic Z0011 populations.

#### 7.1.8 Future directions: liquid biopsy and AI risk stratification

Emerging technologies such as circulating tumor DNA (ctDNA) and machine learning models (e.g., Memorial Sloan Kettering’s nomogram) (47) may further refine patient selection, identifying ultra-low-risk cohorts where SLNB can be safely omitted.

### 7.2 Omitting axillary lymph node dissection in sentinel node-positive disease

#### 7.2.1 ACOSOG Z0011 trial: a paradigm shift

The ACOSOG Z0011 trial (Giuliano et al., 2011, 2017) was a landmark study that challenged the necessity of ALND in patients with 1–2 positive sentinel lymph nodes (SLNs). The trial enrolled women with T1–2 tumors and clinically node-negative disease undergoing BCT with whole-breast radiotherapy (RT). At 10 years, there was no significant difference in overall survival (86.3% vs 83.6%) or axillary recurrence (0.5% vs 0.3%) between the SLNB-alone and ALND groups (48). Despite these compelling results, real-world adoption has been gradual. A 2023 meta-analysis found that only 60% of eligible patients avoided ALND, with surgeon experience and institutional protocols being key determinants. Additionally, the trial’s exclusion of mastectomy patients has led to ongoing debate about whether its findings can be extrapolated to this population.

#### 7.2.2 AMAROS trial: radiotherapy as an alternative to ALND

The AMAROS trial (EORTC 10981–22023) was designed to determine whether axillary radiotherapy (ART) could safely replace axillary lymph node dissection (ALND) in patients with a positive sentinel lymph node. With the release of the mature 10-year follow-up, the trial continues to support ART as an effective strategy for regional control, while highlighting important differences in long-term toxicity profiles.

Per the intention-to-treat analysis, axillary recurrence remained rare in both groups at 10 years. The cumulative incidence of axillary recurrence was 0.93% (95% CI, 0.18–1.68; seven events) after ALND compared with 1.82% (95% CI, 0.74–2.94; eleven events) after ART (HR 1.71; 95% CI, 0.67–4.39). Despite the small numerical difference, the absolute rates were low and not statistically different, confirming that ART provides regional control comparable to ALND.

Long-term survival outcomes were also similar. The 10-year overall survival (OS) was 84.6% (95% CI, 81.5–87.1) in the ALND arm versus 81.4% (95% CI, 77.9–84.4) in the ART arm (HR 1.17; 95% CI, 0.89–1.52; *P* = .26). The 10-year disease-free survival (DFS) was 75.0% (95% CI, 71.5–78.2) with ALND and 70.1% (95% CI, 66.2–73.6) with ART (HR 1.19; 95% CI, 0.97–1.46; *P* = .11). These findings reaffirm that omission of ALND does not compromise long-term oncologic outcomes.

Toxicity outcomes clearly favored ART. Updated analyses—including an additional 175 patients—demonstrated consistently higher lymphedema rates with ALND at all assessed time points. At 5 years, lymphedema occurred in 24.5% of patients after ALND compared with 11.9% after ART (*P* <.001). When using patient-reported outcomes across the entire follow-up period, 44.2% of ALND patients reported lymphedema at any point versus 28.6% in the ART group, confirming the persistent morbidity associated with surgical axillary clearance. Quality-of-life outcomes did not differ significantly through 5 years.

However, the long-term follow-up also raised considerations regarding radiation-associated toxicities. While overall rates remained low, ART was associated with a higher 10-year cumulative incidence of second primary malignancies (12.1% after ART vs 8.3% after ALND). Moreover, an external 2022 report observed a 1.5% incidence of angiosarcoma among ART-treated patients, emphasizing the need for ongoing vigilance and careful selection, particularly in patients with left-sided disease where cardiovascular exposure may be a concern (49).

Taken together, the 10-year AMAROS results support ART as an effective and less morbid alternative to ALND for patients with limited nodal disease, while underscoring the importance of balancing long-term radiation risks against surgical morbidity in shared decision-making.

However, concerns persist regarding long-term radiation toxicity, particularly cardiovascular risks in left-sided tumors and the potential for secondary malignancies. A 2022 follow-up study (50) reported a 1.5% incidence of angiosarcoma in the ART group, underscoring the need for careful patient selection.

#### 7.2.3 SENOMAC and POSNOC: expanding de-escalation to mastectomy patients

##### 7.2.3.1 SENOMAC trial

The SENOMAC trial (51) enrolled patients with clinically node-negative (cN0) breast cancer who had one or two sentinel-lymph-node (SLN) macrometastases and then either underwent completion axillary lymph-node dissection (ALND) or omitted further axillary surgery. Importantly, this trial extended eligibility to include patients undergoing mastectomy (in contrast to many prior studies restricted to breast-conservation), those with T3 tumors or extracapsular extension. According to the 2024 NEJM publication the key findings were:

- The estimated 5-year recurrence-free survival (RFS) was 89.7% (95% CI 87.5–91.9) in the SLN-biopsy only arm and 88.7% (95% CI 86.3–91.1) in the ALND arm; the hazard ratio (HR) for recurrence or death was 0.89 (95% CI 0.66–1.19), which met the non-inferiority margin.
- Axillary recurrence remained extremely rare in both groups (exact numbers not detailed in the abstract).
- Subgroup analyses, including patients undergoing mastectomy, showed consistent results supporting omission of ALND in selected patients.

Thus, the SENOMAC trial supports the feasibility of omitting ALND in a broader population—including mastectomy patients—with low nodal burden and modern adjuvant therapy.

##### 7.2.3.2 POSNOC trial

The POSNOC trial (52) randomized women with T1-T2 invasive breast cancer and one or two SLN macrometastases to either adjuvant therapy alone (i.e., systemic therapy ± whole-breast/chest-wall radiotherapy but *no* axillary surgery or nodal RT) or adjuvant therapy plus axillary treatment (ALND or axillary radiotherapy). While full long-term results are pending in a peer-reviewed publication, the trial design and preliminary data allow the following summary:

• The hypothesis is that systemic therapy (along with breast/chest-wall RT where indicated) can compensate for omission of axillary surgery or nodal RT in patients with low-volume nodal disease.

• Earlier interim data suggested non-inferiority for omission of further axillary treatment, though full 5-year axillary recurrence data were awaited.

Given the design and the emerging landscape, POSNOC adds to the evidence that axillary de-escalation may be safely extended beyond the most favorable scenarios.

Together, SENOMAC and POSNOC broaden the de-escalation paradigm from patients undergoing breast-conservation (as in earlier trials) into patients undergoing mastectomy and those with slightly higher risk features (T3, extracapsular extension).

• These data reinforce the principle that systemic therapy and optimized radiotherapy permit reduction of surgery in the axilla without compromising short-term outcomes, at least among carefully selected patients with limited sentinel nodal involvement.

• Key caveats remain: longer follow-up is needed (especially beyond 5 years) to confirm durable axillary control and survival equivalence; patient selection remains critical—low nodal burden, favorable biologic features, and adherence to systemic/radiotherapy guidelines matter.

• For mastectomy patients specifically, the decision whether to omit ALND must consider post-mastectomy radiotherapy (PMRT) protocols, chest-wall + regional node RT policies, and individual risk of locoregional recurrence.

• Ongoing monitoring for late toxicities (e.g., lymphedema, radiation-related effects) and ensuring multidisciplinary coordination remain essential.

#### 7.2.4 Ongoing trials: ALLIANCE A11202 and TAXIS

The ALLIANCE trial (53) (A011202/sometimes cited as A11202) is a randomized phase-III study testing whether definitive axillary radiotherapy can substitute for axillary lymph-node dissection (ALND) in patients with residual nodal disease after neoadjuvant chemotherapy. The trial compares ALND plus regional nodal radiation (excluding the dissected axilla) versus axillary-directed radiotherapy to the undissected axilla and regional nodes, with invasive-breast-cancer recurrence-free interval as the primary endpoint. This study addresses a population historically excluded from de-escalation trials — patients with residual pathologic nodal disease after NAC — and its results are expected to directly inform whether radiation can be used as an effective, less-morbidity alternative to completion ALND in this setting. Mature randomized outcomes are awaited.

##### 7.2.4.1 The TAXIS program tests a complementary de-escalation strategy for clinically node-positive disease

Tailored axillary surgery (TAS) (removal of the clipped/biopsy-proven node, sentinel nodes, and any palpable suspicious nodes) followed by axillary radiotherapy, with or without completion ALND depending on randomization. The published preplanned feasibility substudy (54) and related reports confirm that TAS reliably retrieves the clipped node (clip retrieval ~94.3%) and turns many clinically positive axillae into clinically negative ones. In patients who underwent subsequent ALND, the false-negative rate of TAS was low (≈2.6%), and the procedure removed a median of ~5 nodes (IQR 3–7) when nodal disease was present. Early oncologic and safety signals have been reassuring: short-term axillary recurrence in series of targeted/TAS-based strategies has been very low (reported rates in prospective registries and feasibility cohorts are generally below ~2% at 2–3 years), and TAS-based approaches are consistently associated with substantially lower rates of lymphedema and arm morbidity compared with completion ALND. These data support TAS as a plausible technique to reduce surgical morbidity while maintaining excellent early regional control; definitive noninferiority results from the randomized TAXIS phase-III comparison are awaited to establish this as a new standard.

##### 7.2.4.2 Bottom line for clinicians and guideline writers

ALLIANCE A011202 and TAXIS are complementary trials testing radiation-based and surgery-sparing strategies for higher-risk or node-positive patients respectively. The feasibility data from TAXIS and contemporary targeted-dissection registries provide encouraging short-term oncologic safety and clear reductions in lymphedema, but both randomized trials remain the definitive tests; their mature results are needed before broad adoption outside clinical trials.

##### 7.2.4.3 (SINODAR ONE + NEONOD 2 )

The SINODAR ONE trial (55) provides level I evidence that axillary lymph node dissection (ALND) may be safely omitted in T1–T2 tumors with ≤2 positive sentinel nodes, even in post-mastectomy settings. Similarly, the ongoing NEONOD 2 trial (56) is evaluating the omission of ALND in patients who, after NACT, show only micrometastases (pN1mi) in sentinel nodes. Together, these studies reinforce the global shift toward minimizing axillary morbidity without compromising oncologic outcomes.

## 8 Clinical implementation and unanswered questions

### 8.1 Barriers to adoption

**Figure 2:**
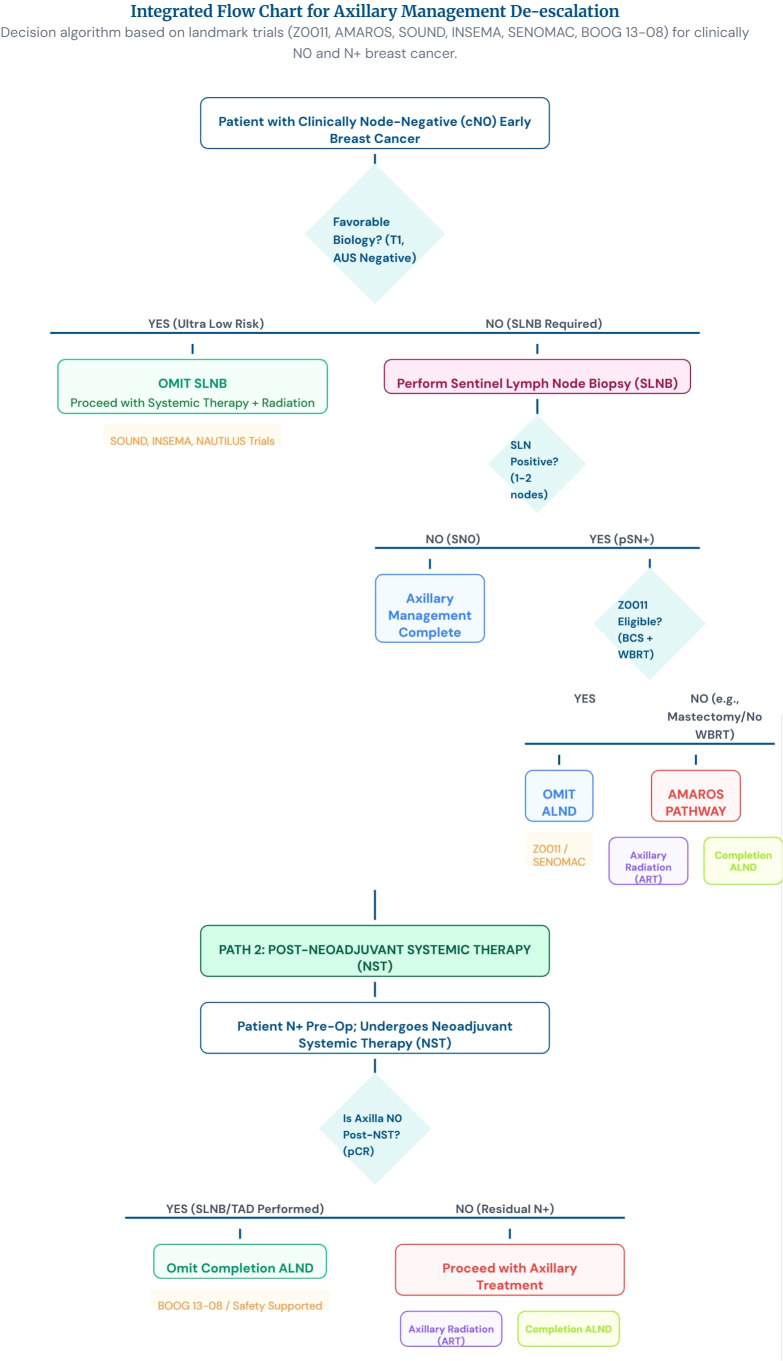
Integrated flow chart of axillary managment de-esclation.

- Surgeon hesitation: 40% of eligible patients still receive ALND due to training biases.
- Global disparities: <30% of low-income countries have SLNB access (**Figure 2**).

### 8.2 Future research priorities

#### 8.2.1 Long-term recurrence outcomes after SLNB omission

Although contemporary trials consistently show excellent 5–10-year regional control when ALND is omitted in patients with minimal sentinel-node involvement, true long-term data are still lacking. Extended follow-up beyond 20 years will be essential to understand late regional failures, particularly among younger patients, those with hormone receptor–positive tumors prone to late relapse, and individuals treated with modern systemic regimens.

#### 8.2.2 Integration of immunotherapy to enable further surgical de-escalation

As neoadjuvant immunotherapy becomes standard for high-risk breast cancer subtypes—such as TNBC treated with pembrolizumab in KEYNOTE-522 (57)—future studies must determine whether higher nodal pCR rates can justify additional reductions in axillary surgery or radiotherapy. Approaches that combine immunotherapy with targeted axillary techniques, including selective removal of clipped nodes, may help define the limits of safe de-escalation.

#### 8.2.3 Refining axillary management after neoadjuvant therapy, particularly in patients initially presenting with cN+ disease

Emerging data from the I-SPY2 platform suggest that axillary surgery may be safely reduced without compromising regional control, even in patients who were node-positive at diagnosis. The recent analysis by Boughey et al. (2025), involving more than 1, 500 participants, demonstrated that response-adapted strategies using clipped-node sampling and targeted axillary dissection can achieve excellent oncologic outcomes post-NAC. These findings highlight the potential for omitting completion ALND in patients who achieve nodal pCR.

#### 8.2.4 Optimizing the use of targeted axillary dissection versus SLNB alone

With widespread adoption of clip placement in biopsy-proven nodes and reliable image-guided retrieval after neoadjuvant therapy, further work is needed to clarify when TAD should replace SLNB, whether the two techniques offer complementary benefits, and how each affects false-negative rates, lymphedema risk, and long-term regional recurrence.

#### 8.2.5 Developing biologic and imaging biomarkers to improve patient selection

Future de-escalation efforts will benefit from markers that predict nodal response with high accuracy. Molecular profiling, radiomic signatures, and advances in MRI or contrast-enhanced mammography may ultimately provide individualized risk stratification, helping clinicians identify which patients can safely avoid ALND or axillary radiotherapy—especially within higher-risk biologic subtypes (55).

## 9 Conclusion

De-escalation is safe in carefully selected patients, but precision is paramount. Integration of genomic profiling (Oncotype, MammaPrint, ctDNA), systemic therapy advances (CDK4/6 inhibitors, checkpoint inhibitors), and AI-driven risk models will refine patient selection, enabling broader safe omission of axillary surgery and potentially breast surgery in exceptional responders. Multidisciplinary collaboration and patient-centered decision-making remain essential to overcoming adoption barriers and ensuring equitable global access.

## References

[1] Halsted WS . The results of operations for cure of cancer of the breast performed at the Johns Hopkins Hospital from June 1889 to January 1894. Ann Surg. (1894) 20:497–555. doi: 10.1097/00000658-189407000-00075, PMID: 17860107 PMC1493925

[2] Halsted CP Benson JR Jatoi I . A historical account of breast cancer surgery: beware of local recurrence but be not radical. Future Oncol. (2014) 10:1649–57. doi: 10.2217/fon.14.98, PMID: 25145433

[3] National Institutes of Health Consensus Development Panel . Consensus statement: treatment of early-stage breast cancer. J Natl Cancer Inst Monogr. (1992) 11):1–5. 1627416

[4] Sakorafas GH Safioleas M . Breast cancer surgery: a historical narrative. Part II. 18th and 19th centuries. Eur J Cancer Care (Engl). (2010) 19:6–29. doi: 10.1111/j.1365-2354.2009.01020.x, PMID: 19674073

[5] Plesca M Bordea C El Houcheimi B Ichim E Blidaru A . Evolution of radical mastectomy for breast cancer. J Med Life. (2016) 9:183–6., PMID: 27453752 PMC4863512

[6] Patey DH Dyson WH . The prognosis of carcinoma of the breast in relation to the type of operation performed. Br J Cancer. (1948) 2:7–13. doi: 10.1038/bjc.1948.2, PMID: 18863724 PMC2007539

[7] Madden JL . Modified radical mastectomy. Surg Gynecol Obstet. (1965) 121:1221–30. 5851617

[8] Fisher B . Laboratory and clinical research in breast cancer—a personal adventure: the David A. Karnofsky memorial lecture. Cancer Res. (1980) 40:3863–74. 7008932

[9] Fisher ER Wang J Bryant J Fisher B Mamounas E Wolmark N . Pathobiology of preoperative chemotherapy. Cancer. (2002) 95:681–95. doi: 10.1002/cncr.10741, PMID: 12209710

[10] Abdel-Razeq H Marei L Saadeh SS Abdulelah H Abu-Nasser M Salam M . From clinical trials to clinical practice: outcome of NSABP-B27 neoadjuvant chemotherapy regimen for high-risk early-stage breast cancer. Breast Cancer Res Treat. (2017) 165:771–7. doi: 10.1007/s10549-017-4359-5, PMID: 28667456

[11] Amalric R Santamaria F Robert F . Conservation therapy of operable breast cancer: results at 5, 10, and 15 years in 2216 consecutive cases. In: Harris JP Hellman S Silen W , editors. Conservative Management of Breast Cancer. Lippincott, Philadelphia, PA (1983). p. 15–21.

[12] Calle R Vilcoq JR Pilleron JP . Conservative treatment of operable breast carcinoma by irradiation with or without limited surgery: ten years results. In: Harris JR Hellman S Silen W , editors. Conservative Management of Breast Cancer. Lippincott, Philadelphia, PA (1983). p. 3–9.

[13] Recht A Silver B Schnitt S Connolly J Hellman S Harris JR . Breast relapse following primary radiation therapy for early breast cancer. I. Classification, frequency, and salvage. Int J Radiat Oncol Biol Phys. (1985) 11:1271–6. doi: 10.1016/0360-3016(85)90203-7, PMID: 4008287

[14] Montague ED Schell SR Romsdahl MM . Conservative surgery and irradiation in clinically favorable breast cancer: the MD Anderson experience. In: Harris JR Hellman S Silen W , editors. Conservative Management of Breast Cancer. Lippincott, Philadelphia, PA (1983). p. 53–9.

[15] Arriagada R Lê MG Rochard F Contesso G . Conservative treatment versus mastectomy in early breast cancer: patterns of failure with 15 years of follow-up data. Institut Gustave-Roussy Breast Cancer Group. J Clin Oncol. (1996) 14:1558–64. doi: 10.1200/JCO.1996.14.5.1558, PMID: 8622072

[16] Fisher B Anderson S Bryant J Margolese RG Deutsch M Fisher ER . Twenty-year follow-up of a randomized trial comparing total mastectomy, lumpectomy, and lumpectomy plus irradiation for the treatment of invasive breast cancer. N Engl J Med. (2002) 347:1233–41. doi: 10.1056/NEJMoa022152, PMID: 12393820

[17] Veronesi U Banfi A Salvadori B . Local control and survival in early breast cancer: the Milan trial. Int J Radiat Oncol Biol Phys. (1986) 12:717–20. doi: 10.1016/0360-3016(86)90027-1, PMID: 3519549

[18] Boughey JC Rosenkranz KM Ballman KV McCall L Haffty BG Cuttino LW . Abstract GS4-01: impact of breast conservation therapy on local recurrence in patients with multiple ipsilateral breast cancer—results from ACOSOG Z11102 (Alliance). Cancer Res. (2023) 83:GS4–01. doi: 10.1158/1538-7445.SABCS22-GS4-01 PMC10256355 36977292

[19] Boughey JC Rosenkranz KM Ballman KV McCall L Haffty BG Cuttino LW . Local recurrence after breast-conserving therapy in patients with multiple ipsilateral breast cancer: results from ACOSOG Z11102 (Alliance). J Clin Oncol. (2023) 41:3184–93. doi: 10.1200/JCO.22.02553, PMID: 36977292 PMC10256355

[20] Rosenkranz KM Ballman K McCall L McCarthy C Kubicky CD Cuttino L . Cosmetic outcomes following breast-conservation surgery and radiation for multiple ipsilateral breast cancer: data from the Alliance Z11102 study. Ann Surg Oncol. (2020) 27:4650–61. doi: 10.1245/s10434-020-08893-w, PMID: 32699926 PMC7554157

[21] Nos C Bourgeois D Darles C Asselain B Campana F Zafrani B . Conservative treatment of multifocal breast cancer: a comparative study. Bull Cancer. (1999) 86:184–8., PMID: 10066949

[22] Kaplan J Giron G Tartter PI Bleiweiss IJ Estabrook A Smith SR . Breast conservation in patients with multiple ipsilateral synchronous cancers. J Am Coll Surg. (2003) 197:726–9. doi: 10.1016/S1072-7515(03)00528-7, PMID: 14585405

[23] Oh JL Dryden MJ Woodward WA Yu TK Tereffe W Strom EA . Locoregional control of clinically diagnosed multifocal or multicentric breast cancer after neoadjuvant chemotherapy and locoregional therapy. J Clin Oncol. (2006) 24:4971–5. doi: 10.1200/JCO.2006.06.4578 17075114

[24] Gentilini O Botteri E Rotmensz N Da Lima L Caliskan M Garcia-Etienne CA . Conservative surgery in patients with multifocal/multicentric breast cancer. Breast Cancer Res Treat. (2009) 113:577–83. doi: 10.1007/s10549-008-9950-8, PMID: 18330695

[25] Lynch SP Lei X Hsu L Meric-Bernstam F Buchholz TA Zhang H . Breast cancer multifocality and multicentricity and locoregional recurrence. Oncologist. (2013) 18:1167–73. doi: 10.1634/theoncologist.2013-0184, PMID: 24136008 PMC3825299

[26] Wolters R Wöckel A Janni W Wischnewsky MB Buchholz S Battista MJ . Comparing the outcome between multicentric and multifocal breast cancer: what is the impact on survival, and is there a role for guideline-adherent adjuvant therapy? A retrospective multicenter cohort study of 8935 patients. Breast Cancer Res Treat. (2013) 142:579–90. doi: 10.1007/s10549-013-2760-0, PMID: 24258258

[27] Winters ZE Horsnell J Elvers KT Maxwell AJ Jones LJ Shaaban AM . Systematic review of the impact of breast-conserving surgery on cancer outcomes of multiple ipsilateral breast cancers. BJS Open. (2018) 2:162–74. doi: 10.1002/bjs5.64, PMID: 30079385 PMC6069349

[28] University College London . MIAMI safe surgery for multiple breast cancers (ClinicalTrials.gov identifier NCT03514654) (2023). ClinicalTrials.gov.

[29] Meyers AK Al-Saffar F Saffari AM Wei S Strawderman MS Zhang S . Omission of breast surgery in exceptional responders after neoadjuvant chemotherapy—what are future possibilities?—a narrative review. Transl Breast Cancer Res. (2024) 5:65. doi: 10.21037/tbcr-24-65, PMID: 40421157 PMC12104956

[30] van Loevezijn AA van der Noordaa ME van Werkhoven ED Loo CE Winter-Warnars GA Wiersma T . Minimally invasive complete response assessment of the breast after neoadjuvant systemic therapy for early breast cancer (MICRA trial): interim analysis of a multicenter observational cohort study. Ann Surg Oncol. (2021) 28:3243–53. doi: 10.1245/s10434-020-09273-0, PMID: 33263830 PMC8119397

[31] Kuerer HM Smith BD Krishnamurthy S Yang WT Valero V Shen Y . Eliminating breast surgery for invasive breast cancer in exceptional responders to neoadjuvant systemic therapy: a multicentre, single-arm, phase 2 trial. Lancet Oncol. (2022) 23:1517–24. doi: 10.1016/S1470-2045(22)00613-1, PMID: 36306810

[32] Parsons HA Blewett T Chu X Cohen O Helvie K Nayar U . Circulating tumor DNA association with residual cancer burden after neoadjuvant chemotherapy in triple-negative breast cancer in TBCRC 030. medRxiv. (2023). doi: 10.1101/2023.03.06.23286772, PMID: 37597579 PMC10898256

[33] Basik M Cecchini RS De Los Santos JF Umphrey HR Julian TB Mamounas EP . Breast tumor-Bed biopsy for pathological complete response prediction: the NRG-BR005 nonrandomized clinical trial. JAMA Surgery. (2025) 160:723–31. doi: 10.1001/jamasurg.2025.1072, PMID: 40332918 PMC12060017

[34] Braunstein LZ Mitchell M Bandos H Sikov WM Khan AJ Chen PY . A phase III randomized trial of radiotherapy optimization for low-risk HER2-positive breast cancer (HERO): NRG-BR008. J Clin Oncol. (2024) 42:TPS613. doi: 10.1200/JCO.2024.42.16_suppl.TPS613, PMID: 40574381 PMC12218515

[35] Kim T Giuliano AE Lyman GH . Lymphatic mapping and sentinel lymph node biopsy in early-stage breast carcinoma. Cancer. (2006) 106:4–16. doi: 10.1002/cncr.21568, PMID: 16329134

[36] Julian TB Anderson SJ Krag DN Harlow SP Costantino JP Ashikaga T . Ten-year follow-up results of NSABP B-32, a randomized phase III clinical trial to compare sentinel node resection with conventional axillary dissection in clinically node-negative breast cancer patients. J Clin Oncol. (2013) 31:1000. doi: 10.1200/jco.2013.31.15_suppl.1000

[37] Gentilini OD Botteri E Sangalli C Galimberti V Porpiglia M Agresti R . Sentinel lymph node biopsy vs no axillary surgery in patients with small breast cancer and negative results on ultrasonography of axillary lymph nodes: the SOUND randomized clinical trial. JAMA Oncol. (2023) 9:1557–64. doi: 10.1001/jamaoncol.2023.3759, PMID: 37733364 PMC10514873

[38] Gentilini OD . Lessons from the SOUND trial and future perspectives on axillary staging in breast cancer. Br J Surg. (2024) 111:znad391. doi: 10.1093/bjs/znad391, PMID: 38059555 PMC10771253

[39] Reimer T Hartmann S Stachs A Krabisch P Kühn T Thill M . Axillary surgery in breast cancer: primary results of the INSEMA trial. N Engl J Med. (2025) 392:1051–64. doi: 10.1056/NEJMoa2412063, PMID: 39665649

[40] Morrow M . Sentinel-lymph-node biopsy in early-stage breast cancer—is it obsolete? N Engl J Med. (2025) 392:1134–6. doi: 10.1056/NEJMe2414899, PMID: 39665673

[41] Jung JG Ahn SH Lee S Kim EK Ryu JM Park S . No axillary surgical treatment for lymph node-negative patients after ultrasonography (NAUTILUS): protocol of a prospective randomized clinical trial. BMC Cancer. (2022) 22:189. doi: 10.1186/s12885-022-09273-1, PMID: 35184724 PMC8859876

[42] Johnston SRD Harbeck N Hegg R Toi M Martin M Shao ZM . Abemaciclib plus endocrine therapy for hormone receptor-positive, HER2-negative, node-positive, high-risk early breast cancer (monarchE): results from a preplanned interim analysis of a randomised, open-label, phase 3 trial. Lancet Oncol. (2023) 24:77–90. doi: 10.1016/S1470-2045(22)00694-5, PMID: 36493792 PMC11200328

[43] Kalinsky K Barlow WE Meric-Bernstam F Chakravarty D Albain KS Lazar AA . 21-gene assay to inform chemotherapy benefit in node-positive breast cancer. N Engl J Med. (2021) 385:2336–47. doi: 10.1056/NEJMoa2108873, PMID: 34914339 PMC9096864

[44] Sparano JA Gray RJ Makower DF Wood WC Lowery MA Barlow WE . TAILORx: phase III trial of chemoendocrine therapy versus endocrine therapy alone in hormone receptor–positive, HER2-negative, node-negative breast cancer and an intermediate prognosis 21-gene recurrence score. J Clin Oncol. (2018) 36:LBA1. doi: 10.1200/JCO.2018.36.18_suppl.LBA1

[45] van Roozendaal LM Vane ML van Dalen T van der Hage JA Beets-Tan RG Lobbes MB . Clinically node-negative breast cancer patients undergoing breast-conserving therapy: sentinel lymph node procedure versus follow-up (BOOG 2013-08). BMC Cancer. (2017) 17:459. doi: 10.1186/s12885-017-3443-x, PMID: 28668073 PMC5494134

[46] Li J Wang Z Li X Pang L Cao X Zhou J . Feasibility of sentinel lymph node biopsy omission after integration of 18F-FDG dedicated lymph node PET in early breast cancer: a prospective phase II trial. Cancer Biol Med. (2022) 19:1100–8. doi: 10.20892/j.issn.2095-3941.2022.0085, PMID: 40826526 PMC9334763

[47] Van Zee KJ Manasseh DM Bevilacqua JL Boolbol SK Fey JV Tan LK . A nomogram for predicting the likelihood of additional nodal metastases in breast cancer patients with a positive sentinel node biopsy. Ann Surg Oncol. (2003) 10:1140–51. doi: 10.1245/ASO.2003.03.015, PMID: 14654469

[48] Giuliano AE Ballman KV McCall L Beitsch PD Whitworth PW Blumencranz PW . Effect of axillary dissection vs no axillary dissection on 10-year overall survival among women with invasive breast cancer and sentinel node metastasis: the ACOSOG Z0011 (Alliance) randomized clinical trial. JAMA. (2017) 318:918–26. doi: 10.1001/jama.2017.11470, PMID: 28898379 PMC5672806

[49] Bartels SA van de Vijver KK van Dalen T van Amerongen EC van der Hage J Voogd AC . Radiotherapy or surgery of the axilla after a positive sentinel node in breast cancer: 10-year results of the randomized controlled EORTC 10981–22023 AMAROS trial. J Clin Oncol. (2023) 41:2159–65. doi: 10.1200/JCO.22.01565, PMID: 36383926

[50] Rugo HS Singer L . First, do no harm: risk of secondary cancer after breast cancer treatment. Lancet Oncol. (2022) 23:1350–2. doi: 10.1016/S1470-2045(22)00627-1, PMID: 36240804

[51] de Boniface J Filtenborg Tvedskov T Rydén L Szulkin R Reimer T Kühn T . et al. Omitting axillary dissection in breast cancer with sentinel-Node metastases. N Engl J Med. (2024) 390:11631175. doi: 10.1056/NEJMoa2313487, PMID: 38598571

[52] Goyal A Dodwell D . POSNOC: a randomised trial looking at axillary treatment in women with one or two sentinel nodes with macrometastases. Clin Oncol (R Coll Radiol). (2015) 27:692–5. doi: 10.1016/j.clon.2015.07.005, PMID: 26254841

[53] National Cancer Institute . A randomized phase III trial comparing axillary lymph node dissection to axillary radiation in breast cancer patients (cT1–3 N1) who have positive sentinel lymph node disease after neoadjuvant chemotherapy. ClinicalTrials.gov. Identifier NCT01901094. Published July 2013.

[54] Heidinger M Lerner R Geiger M Gentilini O Saccilotto R Tausch C . Tailored axillary surgery – A novel concept for clinically node-positive breast cancer: the OPBC-03/TAXIS trial. The Breast. (2023) 70:173182. doi: 10.1016/j.breast.2023.03.005, PMID: 36922305 PMC10034500

[55] Tinterri C Gentile D Gatzemeier W Sagona A Barbieri E Testori A . Preservation of axillary lymph nodes compared with complete dissection in T1–2 breast cancer patients presenting one or two metastatic sentinel lymph nodes: the SINODAR-ONE multicenter randomized clinical trial. Ann Surg Oncol. (2022) 29:5732–44. doi: 10.1245/s10434-022-11866-w, PMID: 35552930

[56] Tinterri C Gentile D Veronesi U . NEONOD 2: rationale and design of a multicenter non-inferiority trial to assess the effect of axillary surgery omission on the outcome of breast cancer patients presenting only micrometastasis in the sentinel lymph node after neoadjuvant chemotherapy. Contemp Clin Trials Commun. (2019) 17:100496. doi: 10.1016/j.conctc.2019.100496, PMID: 31872159 PMC6909193

[57] Schmid P Cortes J Pusztai L . KEYNOTE-522: phase III study of pembrolizumab plus chemotherapy vs placebo plus chemotherapy as neoadjuvant treatment, followed by pembrolizumab vs placebo as adjuvant treatment for early triple-negative breast cancer (TNBC). Ann Oncol. (2019) 30:v853–4. doi: 10.1093/annonc/mdz394.003

